# Association between an indel polymorphism within the distal promoter of *EGLN2* and cancer risk: An updated meta‐analysis

**DOI:** 10.1002/mgg3.936

**Published:** 2019-08-15

**Authors:** Shulong Zhang, Kaihua Zhu, Zuoliang Zhang, Hui Wang, Xiaolong Wang

**Affiliations:** ^1^ Department of General Surgery Xuhui District Central Hospital of Shanghai Shanghai China

**Keywords:** cancer, *EGLN2*, polymorphism, risk

## Abstract

**Background:**

The association between a 4‐bp indel polymorphism (rs10680577) within the distal promoter of *EGLN2* and cancer risk has been investigated by several case–control studies in recent years, but investigation results were inconsistent. Thus, a systematic assessment of the association was performed based on a literature review and pooled analysis.

**Methods:**

Two investigators independently retrieved relevant studies from PubMed, Chinese National Knowledge Infrastructure (CNKI), Embase, and Google Scholar. The fixed or random effects model was selected to calculate odds ratios (ORs) with 95% confidence intervals (CIs) based on heterogeneity level. All analyses including heterogeneity assessment, subgroup analysis, sensitivity analysis, and publication bias assessment were performed using RevMan 5.3 software and Stata 12.0 software.

**Results:**

A total of six relevant studies with 3,406 cases and 5,147 controls were included in the final analysis. The overall pooled analysis showed that *EGLN2* rs10680577 polymorphism was significantly associated with cancer risk under all genetic models. However, subgroup analysis based on cancer type showed that the polymorphism was significantly associated with the risk of digestive system cancer under all genetic models, and with the risk of lung cancer under dominant model, heterozygote comparison model, and allele comparison model. Subgroup analysis based on population sources showed a significant association in Chinese population under all genetic models.

**Conclusion:**

The present result suggests that *EGLN2* rs10680577 polymorphism is associated with cancer risk, and may act as a promising predictive biomarker for cancer risk, especially in Chinese population. However, further well‐designed studies are warranted to confirm these results.

## 1 INTRODUCTION

Cancer is one of the most common disorders causing considerable mortality. Its etiology is complex and involved in environmental and genetic factors. For genetic factors, polymorphisms within several cancer‐related genes have been shown to affect an individual's susceptibility to cancer (Chen et al., 2018; Gao, Yang, Wang, & Zhang, 2016; Gu et al., 2018; Shi et al., 2017). Among these cancer‐related genes, *EGLN2* (OMIM accession number: 606424) has been gaining great attention (Erez et al., 2003; Xie et al., 2014).

*EGLN2* is located in the chromosome 19q13.2 region, and encodes an enzyme capable of recognizing conserved prolyl residues in the α‐subunit of hypoxia inducible factor (HIF) and hydroxylating it (Pugh, 2016; Schofield, & Ratcliffe, 2004). Subsequently, the hydroxylated HIF are rapidly destroyed via the von Hippel–Lindau protein‐dependent ubiquitination (Jaakkola et al., 2001; Pugh, 2016). Therefore, *EGLN2* plays an important role in regulating the stability and transcriptional activity of HIF. HIF is a transcriptional complex that consists of an oxygen‐dependent α‐subunit and a constitutively expressed beta‐subunit, and involved in the occurrence and development of many types of solid tumors by coordinating the cellular response to hypoxia and oxygen homeostasis (Huang & Lin, 2017; Schito & Semenza, 2016; Tong, Tong, & Liu, 2018). So we speculated that genetic polymorphisms affecting *EGLN2* expression could confer an individual's susceptibility to cancer. Interestingly, several studies have focused on the association between a functional polymorphism within *EGLN2* and the risk of cancers, including breast cancer, lung cancer, colorectal cancer, gastric cancer, and hepatocellular carcinoma (Che et al., 2014; Hashemi, Danesh, et al., 2018; Li et al., 2017; Wang, Zhang, Zhou, Chen, & Yu, 2014; Zhu, Luo, & Li, 2019; Zhu et al., 2012). This functional polymorphism is a 4‐bp insertion/deletion (indel) polymorphism (rs10680577) within the distal promoter of *EGLN2*, which can affect the expression of *EGLN2* (Zhu et al., 2012). Although the role of the functional polymorphism in cancer risk has been reported, the result is ambiguous and needs to be further elucidated. In view of the fact that meta‐analysis is a statistical analysis that has the capacity to contrast results from different studies and identifies sources of disagreement among those results, or other interesting relationships that may come to light in the context of multiple studies, we utilized the method to systematically assess the association of the rs10680577 polymorphism with cancer risk in the present study.

## 2 METHODS

### 2.1 Literature retrieval

Two investigators independently retrieved relevant studies from PubMed, Chinese National Knowledge Infrastructure (CNKI), Embase, and Google Scholar. The last retrieval was updated on 15 January 2019 with the following keywords: “cancer”, “tumor”, “*PHD1*”, “*EGLN2*”, “polymorphism”, “variant”, and “rs10680577”. In addition, references in potential articles were also reviewed in order to obtain more relevant studies.

### 2.2 Inclusion criteria

All articles were reviewed by two investigators independently. Studies were considered eligible if they met the following criteria: (a) investigating the association of *EGLN2* rs10680577 polymorphism and cancer risk; (b) case–control studies; and (c) available genotype frequencies. Meanwhile, the following exclusion criteria were also applied: (a) review, abstracts, case reports, and editorials; (b) studies that did not report genotype frequencies; and (c) studies that reported duplicated results.

### 2.3 Quality score assessment

The Newcastle–Ottawa scale was utilized to assess the quality of studies (Stang, 2010). A total of three categories including selection, comparability, and exposure were used to calculate the quality score of studies. Thereinto comparability was endowed with at most two stars. Other categories were endowed with at most one star. Thus, the highest quality study will have nine stars. A total score of 3 or lower, 4 to 6 and 7 or greater was considered to be of low, medium and high quality, respectively.

### 2.4 Data extraction

Two investigators independently extracted data from included studies according to a standardized form. For each study, the following information was extracted: name of first author, publication year, country, cancer type, genotyping method, sample size, and genotype and allele frequencies. Any disagreements will be resolved by discussing with a third investigator.

### 2.5 Statistical analysis

Statistical analysis was performed using Review Manager 5.3 software and Stata 12.0 software. The pooled odds ratios (ORs) with corresponding 95% confidence intervals (95% CIs) were calculated to assess the strength of the association. Both the pooled ORs and the lower limit of 95% CIs > 1 indicated an increased risk. Both the pooled ORs and the upper limit of 95% CIs < 1 indicated a decreased risk. The following five genetic models were used in this meta‐analysis: dominant model [(Ins/Del + Del/Del) vs. Ins/Ins], recessive model [Del/Del vs. (Ins/Del + Ins/Ins)], homozygote comparison model [Del/Del vs. Ins/Ins], heterozygote comparison model [Ins/Del vs. Ins/Ins], and allele comparison model [Del vs. Ins]. A value of *P*z < .05 was considered as the significance threshold for each genetic model. The Chi‐squared test was conducted to evaluate whether these studies deviated from Hardy–Weinberg equilibrium (HWE), and the threshold for disequilibrium was *P*_HWE_ < .05. Cochran's Q test was performed to assess heterogeneity across individual studies, and *P_H_* ≤ .10 suggested heterogeneity. The fixed effects model was selected to estimate the pooled OR if *P_H_* > .10; otherwise, the random effects model was adopted. Funnel plots and Egger's test were used to assess the publication bias. *P_E_* < .05 indicated significant publication bias.

## 3 RESULTS

### 3.1 Characteristics of included studies

**Figure 1:**
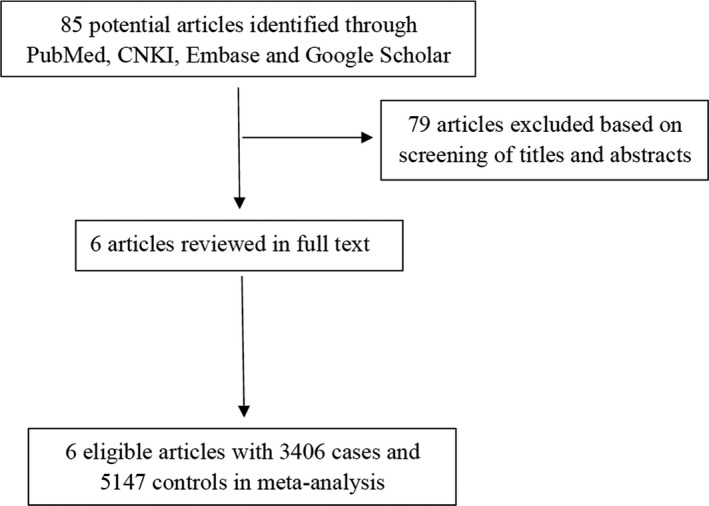
Flow diagram of literature selection

A flow diagram for Literature retrieval strategy is shown in Figure 1. According to the retrieval strategy, 85 articles were identified in the initial retrieval. After reviewing titles and abstracts, 79 articles were excluded and six articles were further reviewed in full text. Based on the criteria of eligible studies, six relevant studies including 3,406 cases and 5,147 controls were used for the final meta‐analysis (Table 1 and Table S1). Among them, three studies focused on digestive system cancer (colorectal cancer, gastric cancer hepatocellular carcinoma), two on lung cancer and one on breast cancer. In addition, all studies were endowed with at least six stars, suggesting that their quality was adaptable (Table S2).

**Table 1:** Characteristics of studies included in the meta‐analysis

First author	Publication year	Country	Cancer type	Genotyping method	Case	Control	Association with cancer risk
Mohammad Hashemi	2018	Iran	Breast cancer	PCR‐RFLP	134	154	No
Jing Zhu	2018	China	Lung cancer	PAGE	376	419	Yes
Chaoyang Li	2017	China	Colorectal cancer	PAGE	1,008	1,240	Yes
Jian Wang	2014	China	Gastric cancer	PAGE	415	830	Yes
Jianhua Che	2014	China	NSCLC	PAGE	406	812	Yes
Zhansheng Zhu	2012	China	Hepatocellular carcinoma	PAGE	1,067	1,692	Yes

Abbreviations: NSCLC, non‐small cell lung cancer; PAGE, Polyacrylamide gel electrophoresis; PCR‐RFLP, PCR‐restriction fragment length polymorphism.

### 3.2 Meta‐analysis results

**Figure 2:**
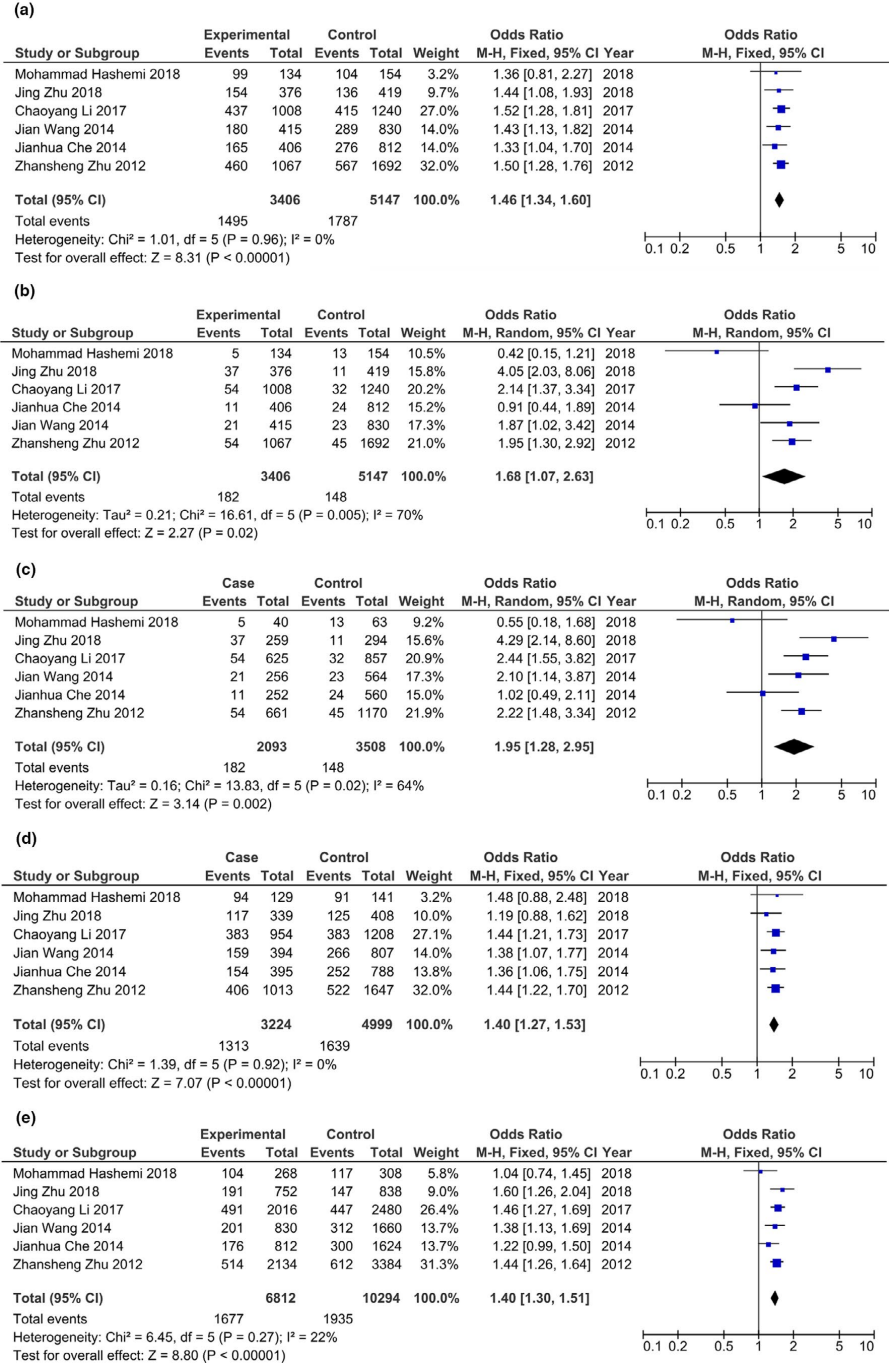
Forest plots for the associations between *EGLN2* rs10680577 polymorphism and cancer risk in the overall population (a: dominant model; b: recessive model; c: homozygote comparison model; d: heterozygote comparison model; e: allele comparison model)

As shown in Table 2, the overall pooled analysis showed that *EGLN2* rs10680577 polymorphism was significantly associated with cancer risk under all genetic models [(Ins/Del + Del/Del) vs. Ins/Ins:OR = 1.46, 95% CI = 1.34–1.60, *P*_Z_ < .001; Del/Del vs. (Ins/Del + Ins/Ins): OR = 1.68, 95% CI = 1.07–2.63, *P*_Z_ = .02; Del/Del vs. Ins/Ins: OR = 1.95, 95% CI = 1.28–2.95, *P*_Z_ = .002; Ins/Del vs. Ins/Ins: OR = 1.40, 95% CI = 1.27–1.53, *P*_Z_ < .001; Del vs. Ins: OR = 1.40, 95% CI = 1.30–1.51, *P*_Z_ < .001] (Figure 2). Subgroup analysis based on cancer type showed that *EGLN2* rs10680577 polymorphism was significantly associated not only with the risk of digestive system cancer under all genetic models [(Ins/Del + Del/Del) vs. Ins/Ins:OR = 1.50, 95% CI = 1.35–1.66, *P_Z_* < .001; Del/Del vs. (Ins/Del + Ins/Ins): OR = 2.00, 95% CI = 1.53–2.62, *P*_Z_ < .001; Del/Del vs. Ins/Ins: OR = 2.28, 95% CI = 1.74–2.99, *P*_Z_ < .001; Ins/Del vs. Ins/Ins: OR = 1.43, 95% CI = 1.28–1.59, *P*_Z_ < .001; Del vs. Ins: OR = 1.44, 95% CI = 1.32–1.57, *P*_Z_ < .001], but also with the risk of lung cancer under dominant model [(Ins/Del + Del/Del) vs. Ins/Ins: OR = 1.38, 95% CI = 1.14–1.66, *P*_Z_ < .001], heterozygote comparison model [Ins/Del vs. Ins/Ins: OR = 1.29, 95% CI = 1.06–1.57, *P*_Z_ = .01] and allele comparison model [Del vs. Ins: OR = 1.39, 95% CI = 1.07–1.81, *P*_Z_ = .02]. Subgroup analysis based on population sources showed a significant association in Chinese population under all genetic models [(Ins/Del + Del/Del) vs. Ins/Ins: OR = 1.47, 95% CI = 1.34–1.61, *P*_Z_ < .001; Del/Del vs. (Ins/Del + Ins/Ins): OR = 1.98, 95% CI = 1.38–2.85, *P*_Z_ < .001; Del/Del vs. Ins/Ins: OR = 2.22, 95% CI = 1.56–3.16, *P*_Z_ < .001; Ins/Del vs. Ins/Ins: OR = 1.40, 95% CI = 1.27–1.53, *P*_Z_ < .001; Del vs. Ins: OR = 1.42, 95% CI = 1.32–1.53, *P*_Z_ < .001].

**Table 2:** Summary of the association between *EGLN2* rs10680577 polymorphism and cancer risk

Genetic model	Subgroup	Case/Control	*P_H_*	Effect model	OR (95% CI)	*P*_Z_	*P_E_*
Dominant model [(Ins/Del + Del/Del) vs. Ins/Ins]	Overall	3,406/5,147	.96	Fixed	1.46 (1.34–1.60)	< .001	.394
	Digestive system cancer	2,490/3,762	.92	Fixed	1.50 (1.35–1.66)	< .001	
	Lung cancer	782/1,231	.67	Fixed	1.38 (1.14–1.66)	< .001	
	Breast cancer	134/154	—	—	1.36 (0.81–2.27)	.24	
	China	3,272/4,993	.92	Fixed	1.47 (1.34–1.61)	< .001	
	Iran	134/154	—	—	1.36 (0.81–2.27)	.24	
Recessive model [Del/Del vs. (Ins/Del + Ins/Ins)]	Overall	3,406/5,147	.005	Random	1.68 (1.07–2.63)	.02	.263
	Digestive system cancer	2,490/3,762	.93	Fixed	2.00 (1.53–2.62)	< .001	
	Lung cancer	782/1,231	.003	Random	1.93 (0.45–8.33)	.38	
	Breast cancer	134/154	—	—	0.42 (0.15–1.21)	.11	
	China	3,272/4,993	.07	Random	1.98 (1.38–2.85)	< .001	
	Iran	134/154	—	—	0.42 (0.15–1.21)	.11	
Homozygote comparison model [Del/Del vs. Ins/Ins]	Overall	2,093/3,508	.02	Random	1.95 (1.28–2.95)	.002	.265
	Digestive system cancer	1,542/2,591	.92	Fixed	2.28 (1.74–2.99)	< .001	
	Lung cancer	511/854	.005	Random	2.10 (0.51–8.61)	.30	
	Breast cancer	40/63	—	—	0.55 (0.18–1.68)	.29	
	China	2,053/3,445	.09	Random	2.22 (1.56–3.16)	< .001	
	Iran	40/63	—	—	0.55 (0.18–1.68)	.29	
Heterozygote comparison model [Ins/Del vs. Ins/Ins]	Overall	3,224/4,999	.92	Fixed	1.40 (1.27–1.53)	< .001	.406
	Digestive system cancer	2,361/3,662	.94	Fixed	1.43 (1.28–1.59)	< .001	
	Lung cancer	734/1,196	.52	Fixed	1.29 (1.06–1.57)	.01	
	Breast cancer	129/141	—	—	1.48 (0.88–2.48)	.14	
	China	3,095/4,858	.85	Fixed	1.40 (1.27–1.53)	< .001	
	Iran	129/141	—	—	1.48 (0.88–2.48)	.14	
Allele comparison model [Del vs. Ins]	Overall	3,406/5,147	.27	Fixed	1.40 (1.30–1.51)	< .001	.354
	Digestive system cancer	2,490/3,762	.90	Fixed	1.44 (1.32–1.57)	< .001	
	Lung cancer	782/1,231	.10	Random	1.39 (1.07–1.81)	.02	
	Breast cancer	134/154	—	—	1.04 (0.74–1.45)	.84	
	China	3,272/4,993	.52	Fixed	1.42 (1.32–1.53)	< .001	
	Iran	134/154	—	—	1.04 (0.74–1.45)	.84	

### 3.3 Sensitivity analysis and publication bias assessment

Sensitivity analysis was performed by excluding one study at a time and subsequently recalculating the overall effect. The result showed that after removing Zhu ZS's study, Wang J’s study, Li CY’s study, or Zhu J’s study, no significant association was found between *EGLN2* rs10680577 polymorphism and cancer risk under recessive genetic model (Table 3), suggesting that results of the overall pooled analysis were not sufficiently robust under recessive genetic model, which might be due to the small number of studies and needed to be further confirmed by large‐scale and well‐designed case–control studies.

**Table 3:** Sensitivity analysis of the overall pooled studies under recessive genetic model

Omitted study	*P_H_*	Effect model	OR (95% CI)	*P*_Z_
Hashemi M's study	.07	Random	1.98 (1.32–2.85)	< .001
Zhu J's study	.03	Random	1.46 (0.94–2.26)	.09
Li CY's study	.003	Random	1.55 (0.86–2.77)	.14
Wang J's study	.002	Random	1.61 (0.93–2.80)	.09
Che JH's study	.01	Random	1.89 (1.19–2.99)	.007
Zhu ZS's study	.002	Random	1.57 (0.86–2.87)	.14

**Figure 3:**
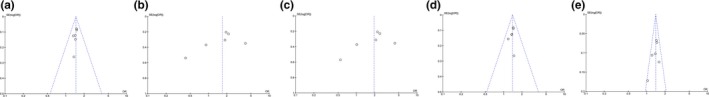
Funnel plots for the association of *EGLN2* rs10680577 polymorphism and cancer risk in the overall population (a: dominant model; b: recessive model; c: homozygote comparison model; d: heterozygote comparison model; e: allele comparison model)

Funnel plots and Egger's test were used to assess the publication bias. As shown in Figure 3, the funnel plots seemed symmetric, suggesting that there was no significant publication bias. In addition, Egger's test also indicated a lack of publication bias (*P_E_* > .05).

## 4 DISCUSSION

In the year 2012, Zhu et al. firstly investigated the association between a 4‐bp indel polymorphism (rs10680577) within the distal promoter of *EGLN2* and cancer risk based on two independent case–control studies, and found that the deletion allele of rs10680577 polymorphism was significantly associated with increased risk of hepatocellular carcinoma. Furthermore, genotype–phenotype correlation studies showed that the deletion allele was significantly correlated with higher expression of *EGLN2* (Zhu et al., 2012). Subsequently, more studies including a meta‐analysis were conducted to explore the association of the rs10680577 polymorphism with the risk of cancer, including lung cancer, gastric cancer, colorectal cancer, and breast cancer (Che et al., 2014; Hashemi, Danesh, et al., 2018; Hashemi, Tabasi, & Ansari, 2018; Li et al., 2017; Wang et al., 2014; Zhu et al., 2019). Thereinto a significant association existed in lung cancer, gastric cancer, and colorectal cancer, which was consistent with the results of Zhu's study in 2012 (Che et al., 2014; Li et al., 2017; Wang et al., 2014; Zhu et al., 2019, 2012). However, there was also an inconsistent result in breast cancer (Hashemi, Danesh, et al., 2018). Hashemi et al. examined the possible association between the rs10680577 polymorphism and the risk of breast cancer in a southeast Iranian population, and did not observe significant differences in the genotype and allele frequencies between breast cancer patients and controls. However, the analysis based on clinicopathological characteristics showed a significant association between the rs10680577 polymorphism and HER2 status. To explain the above inconsistent results, a meta‐analysis including 3,406 cases and 5,147 controls was conducted, and five genetic models were utilized to assess the association between the *EGLN2* rs10680577 polymorphism and cancer risk. The results of our meta‐analysis showed that *EGLN2* rs10680577 polymorphism was significantly associated with cancer risk under all genetic models. However, subgroup analysis based on cancer type showed that *EGLN2* rs10680577 polymorphism was significantly associated with the risk of digestive system cancer under all genetic models, and with the risk of lung cancer under dominant model, heterozygote comparison model, and allele comparison model. No significant association was observed between *EGLN2* rs10680577 polymorphism and the risk of breast cancer. Subgroup analysis based on population sources showed a significant association in Chinese population under all genetic models. No significant association was observed in Iranian population. The emergence of the above inconsistent results may be due to any of the following reasons: (a) a different genetic background between Chinese and Iranian population; (b) a small sample size of the study on Iranian population (only 134 cases and 154 controls); (c) genotype distribution of control samples in Iranian population deviated from HWE.

Compared with previous meta‐analysis, the current meta‐analysis contained more samples and provided more valuable information such as results of subgroup analysis. However, some limitations still existed and needed to be clarified. Firstly, the number of included studies was small and only six case–control studies were analyzed. Secondly, due to insufficient information, potential interactions including gene–gene, gene–environment or gene–some potential covariates were not considered. Thirdly, Literature retrieval strategy was limited by language, and only articles published in English or Chinese were included.

In conclusion, our meta‐analysis determined that the *EGLN2* rs10680577 polymorphism was associated with cancer risk, and may act as a valuable biomarker for predicting cancer risk, especially in Chinese population. However, further well‐designed studies are warranted to confirm these results.

## CONFLICT OF INTEREST

The authors declare no conflict of interest.
