# Supplementary material for: Association between an indel polymorphism within the distal promoter of EGLN2 and cancer risk: An updated meta‐analysis
Source: Mol Genet Genomic Med. 2019 Aug 15;7(10):e00936. doi: 10.1002/mgg3.936 (PMC6785434; doi:10.1002/mgg3.936)
Supplement: Supplementary file 1 [file MGG3-7-e00936-s001.docx]

Table S1. Genotype and allele frequencies of *EGLN2* rs10680577 polymorphism in included studies

|  | Case | | | | | Control | | | | | *P*_HWE_ |
| --- | --- | --- | --- | --- | --- | --- | --- | --- | --- | --- | --- |
| First author | Ins/Ins | Ins/Del | Del/Del | Ins | Del | Ins/Ins | Ins/Del | Del/Del | Ins | Del |  |
| Mohammad Hashemi | 35 | 94 | 5 | 164 | 104 | 50 | 91 | 13 | 191 | 117 | 0.001 |
| Jing Zhu | 222 | 117 | 37 | 561 | 191 | 283 | 125 | 11 | 691 | 147 | 0.52 |
| Chaoyang Li | 571 | 383 | 54 | 1525 | 491 | 825 | 383 | 32 | 2033 | 447 | 0.11 |
| Jian Wang | 235 | 159 | 21 | 629 | 201 | 541 | 266 | 23 | 1348 | 312 | 0.15 |
| Jianhua Che | 241 | 154 | 11 | 636 | 176 | 536 | 252 | 24 | 1324 | 300 | 0.39 |
| Zhansheng Zhu | 607 | 406 | 54 | 1620 | 514 | 1125 | 522 | 45 | 2772 | 612 | 0.09 |
