# Supplementary material for: Association between an indel polymorphism within the distal promoter of EGLN2 and cancer risk: An updated meta‐analysis
Source: Mol Genet Genomic Med. 2019 Aug 15;7(10):e00936. doi: 10.1002/mgg3.936 (PMC6785434; doi:10.1002/mgg3.936)
Supplement: Supplementary file 2 [file MGG3-7-e00936-s002.docx]

Table S2. The quality assessment of all included studies based on the Newcastle-Ottawa Scale.

| Categories | Items | Hashemi M's study | Zhu J's study | Li CY's study | Wang J's study | Che JH's study | Zhu ZS's study |
| --- | --- | --- | --- | --- | --- | --- | --- |
| Selection | Adequacy of case definition | * | * | * | * | - | * |
|  | Representativeness of the cases | * | * | * | * | * | * |
|  | Selection of controls | * | * | * | * | * | * |
|  | Definition of controls | * | * | * | * | * | * |
| Comparability | Comparability of cases/controls | * | * | * | * | * | * |
| Exposure | Ascertainment of exposure | * | * | * | * | * | * |
|  | Same method of ascertainment for cases and controls | * | * | * | * | * | * |
|  | Non-Response rate | - | - | - | - | - | - |
